# Distribution characteristics of FcμR positive cells in small intestinal lymph nodes of Bactrian camel

**DOI:** 10.1371/journal.pone.0287329

**Published:** 2023-07-20

**Authors:** Xie Fei, Jian fei Li, Ying dong Fang, Li ping Liu, Ke Jiang Liu, Wei wei Zeng, Wen hui Wang

**Affiliations:** College of Veterinary Medicine, Gansu Agricultural University, Lanzhou, China; Lerner Research Institute - Cleveland Clinic, UNITED STATES

## Abstract

Exploring the expression characteristics of FcμR in small intestinal lymph nodes of bactrian camels can lay the foundation for further revealing the function of FcμR. The FcμR expression characteristics were systematically analysed by using prokaryotic expression, antibody preparation, immunohistochemical staining and statistical analysis. FcμR positive cells were mainly located in the lymphoid follicles and their numbers decreased in the order of duodenal lymph nodes, jejunal lymph nodes and ileal lymph nodes, and the number of positive cells was statistically significant between different intestinal segments (P<0.05). The FcμR is expressed in lymphoid follicular B cells, which not only facilitates the body’s ability to regulate secretory IgM levels, but also acts as a local immune defence barrier. The small intestine has dual functions of immune tolerance and immune response, the proximal part mainly focuses on immune tolerance, and the distal part mainly focuses on immune response. This distribution ensures the unity of the duodenal absorption and immune defence, and also significantly increases the efficiency of the entire small intestine, which is why the number of FcμR positive cells decreases in the order of duodenal lymph nodes, jejunal lymph nodes and ileal lymph nodes.

## Introduction

In 1986, Sheila Sanders [1] accidentally isolated a single-chain polypeptide from a mouse IgM monoclonal antibody when analyzing B cell activation antigen. It was not until 2009 that people actually identified the gene of this single-chain polypeptide and officially named it FcμR [1–3]. FcμR is a specific receptor of immunoglobulin IgM, and it is also a type I transmembrane protein in the immunoglobulin gene superfamily, mainly composed of extramembrane region, transmembrane region and intracellular region [4]. Unlike chimpanzee, monkey, dog whose intracellular region are composed of tyrosine activation or inhibitory sequence, the intracellular region of Fc°R receptors is composed of conserved serine and tyrosine residues [5–7]. It shows that Fc°R has dual signaling ability, one from the potential adapter protein, non-covalently combined with Fc°R through His residues, the other from the Tyr or SerR residues of the cytoplasmic tail itself [8–10]. Notably, Fc°R is mainly expressed by human B, T and NK cells and mouse B cells, Although both human Fc°R and mouse Fc°R are encoded by a single gene in chromosome 1 and have similar molecular structure, but they are only 54% homologous [11–14]. In terms of biological effects, FcμR mainly plays a role in maintaining humoral immune homeostasis, B cell survival and inhibiting the body producing autoreactive antibodies [15]. Some data show that the secretory IgM levels were significantly higher in FcμR-deficient mice than in wild-type mice, suggesting that some secretory IgM is in a state of binding to the FcμR receptor [16]. When an external pathogen invades, Fc°R will release IgM. Once the content of IgM in serum is greater than that of IgG, Fc°R will induce B cells to secrete IgG, which is the main force of the humoral immunity of the body.

As the central organ of peripheral immune system, lymph nodes are the main residence of mature B cells and the main effect site of the body’s mucosal immune response. When antigen-presenting cells transport external antigens to the lymph nodes, the B cells in lymph nodes are activated, rapidly differentiate into plasma cells and secrete immunoglobulins, and FcμR molecules present on the surface of B cells play an important role in this process [17]. Unfortunately, at present, the research objects of FcμR are only humans and mice. The research sites include blood, bone marrow, tonsil, spleen and appendix. The research methods adopted are mainly Flow cytometric analysis and gene knockout. Morphological exploration of the distribution characteristics of FcμR positive cells in the small intestinal lymph nodes of bactrian camels have not been reported [18–20]. Therefore, the FcμR expression characteristics in small intestine of Bactrian camel were systematically analysed by using prokaryotic expression, antibody preparation, immunohistochemical staining and statistical analysis. We hope that the results of this study will provide support for future exploration of the immunological mechanism of FcμR.

## Materials and methods

### Ethical approval

All experiments were conducted in accordance with the guidelines of Chinese laboratory animal use and care legislation. The animal experiment program was approved by the Animal Welfare and Research Ethics Committee of Gansu Agricultural University Animal Medical College. Approval No:GSAU-AEW-2016-0005

### Materials

New Zealand white rabbits, male, weighing about 2.5 kg, were purchased from the Experimental Animal Center of Lanzhou Veterinary Research Institute in China. Nine healthy young Bactrian camels were purchased from the Minqin County of Gansu Province in China. His Tag protein purification kit (Kit Catalog No.CW0893S) was purchased from Beijing ComWin Biotech in China. HRP conjugated goat anti-rabbit IgG was purchased from Wuhan Boster Biological Technology in China. The expression vector were purchased from Beijing Solarbio Life Sciences and the reference sequence(s) is PET-28a(+) (Kanamycin) in China.

### Gene cloning and vector construction

First, the nucleotide sequence of Bactrian camel FcμR included in National Center for Biotechnology Information was analyzed by bioinformatics analysis software (NCBI Reference Sequence: XM_031438680.1). Then, the nucleotide fragment sequence in the outer region of the membrane was selected and sent to Suzhou GENEWIZ Biotechnology.The fragments was optimised、synthesized and sequenced by Suzhou GENEWIZ Biotechnology. Finally, The recombinant plasmid DNA containing the target gene fragment was produced. The amplification primers were GACTGGTGGACAGCAAATGGGTCGCGGATCCATGCTGAAAGTGATCCCGGAAGTGAA(AA14681-1-1),GGTGGTGGTGCTCGAGTGCGGCCGCAAGCTTTTAATCTTCCATGGCGCTCATGCGGCCCGGA(AA14681-1-18). The amplification conditions were: pre-denaturation: 98°C for 5min, denaturation: 97 °C 18s, annealing: 64°C 20s, elongation:72°C 50s, final extension: 72°C for 2min, denaturation, annealing, and extension cycles were 23. The construction process was as follows: a. The FcμR insert fragment and the PET-28a(+) digestion product linearized with restriction endonucleases BamH1 and Hindlll were connected with T4 enzyme. The product of connection was transformed into Escherichia coli cells, and the transformed bacteria were evenly spread on a plate containing kanamycin antibiotic. Invert the plate and incubate overnight at 37°C. b. Identification of positive clones by colony PCR. Picked individual colonies with a sterile toothpick into 200 μl LB medium and mix well, incubate for 3h at 37°C. c. Select the clone for DNA sequencing. d. Selected the correct clone according to the sequencing results.

### Protein prokaryotic expression

The recombinant plasmid dry powder delivered by Suzhou GENEWIZ Biotechnology was dissolved by 30uL DEPC water vortex, placed on ice, then 3uL recombinant plasmid was added to 50uL BL21 receptor cells (operated in ultra clean bench), after light shakes、ice bath (30min)、heat excitation in 42°C water bath (90s), ice bath 3min and added to 700uL LB liquid culture medium without Kan+ for culture. (the expression vector were purchased from Solarbio Life Sciences and the reference sequence(s) is PET-28a(+) (Kanamycin)). The bacteria were cultured in LB (50 °g/mL Kana) overnight. The expression of the recombinant protein was induced by setting different gradients of inducers and different induction times, and the expressed bacteria were collected for washing and breaking, and the broken bacteria were purified according to the requirements of the His tag protein purification kit (Beijing ComWin Biotech., His-tagged Protein Purification Kit Catalog No.CW0893S).

The purification procedure are as follows: First, the sedimentation was dissolved in Binding Buffer (10 mL and containing urea (8 mol/L)), centrifuged at 4°C for 10 min at 10,000 r/min and the supernatant was collected. Then, the Ni-Agarose Resin packing was mixed and added to the column (1 mL), and after the gel was completely separated from the solution, the ethanol at the bottom of the column was excluded. Finally, the column was equilibrated with 10 mL of Binding Buffer, and the supernatant after centrifugation was filtered through a sterile bacterial filter of 0.45 μm pore size and loaded onto the column, which was placed on a standard-type shaker for overnight shaking. After the solution was layered, the flow-through solution at the bottom of the column was excluded, and the column was immediately washed with 15 mL of Binding Buffer, then the target protein was eluted with Elution Buffer, and the eluate was collected (1mL/tube). Finally, the supernatant, sedimentation were separated for electrophoresis on SDS-PAGE. These proteins were visualized using Coomassie brilliant blue staining.

### Preparation of polyclonal antibodies

The emulsifier used for the first immunisation is Freund’s complete adjuvant and all subsequent immunisations were carried out with Freund’s incomplete adjuvant. The emulsification ratio of both Freund’s complete adjuvant and Freund’s incomplete adjuvant to recombinant protein is always 1:1. The purchased rabbits were fed adaptively for one week, and immunization began after no adverse reactions were observed. The method of multi-point subcutaneous injection on the back and injection to popliteal lymph nodes was used to immunize once every other week, 800 μg each time, and 4 times in total. After the immunization was completed, the rabbits were sacrificed for heart blood collection, and the serum was separated and stored for future use.

### Western blotting (WB)

First, samples of the induced Bactrian camel recombinant protein was prepared, The concentration of the protein was determined by using the BCA kit (Biyuntian Shanghai China P0009) experimental procedure. After conventional SDS-PAGE electrophoresis (15% separating gel and 5% concentrate gel, the voltage was 120 V for the separating gel and 80 V for the stacking gel for electrophoresis), they were transferred to pre-soaked PVDF membranes, electroporated at 100V for 2 hours, and washed 3 times with TBST for 10 minutes each time. Then, the membrane was cut open, placed in 1:400 diluted pre-immune rabbit serum and rabbit antiserum (primary antibody), incubated overnight at 4 degrees, washed 3 times with TBST, each 10min. Finally, a 1:500 dilution of secondary antibody (HRP-labeled goat anti-rabbit IgG) was added, incubated at room temperature for 2 h, washed 3 times with TBST, 10 min each. Add ECL luminescent liquid dropwise, expose and save the picture.

### Enzyme linked immunosorbent assay (ELISA)

Antibody titer was measured by indirect enzyme linked immunosorbent assay (ELISA). The purified protein was diluted to 1–10 μg/mL. The diluted recombinant protein was then diluted again in equal proportions for a total of 8 tubes. Rabbit antiserum was equally diluted from 1:2000, and a total of 10 tubes were diluted. Diluted recombinant protein is coated on plates at 100 μL aliquot per well, 4 °C overnight. The wells were washed three times with PBST buffer. The coated wells were blocked with 5% skimmed milk for 1 h at 37 °C, the wells were washed three times with PBST buffer and then incubated with 100 μL rabbit antiserum with different dilutions. After incubation for 1 h at 37 °C, the wells were incubated with 100 μL HRP-labelled goat anti-rabbit IgG for 1 h at 37 °C after thorough washing. Peroxidase activity on the immunoplate was detected by ophenylenediamine and H_2_O_2_ as enzyme Substrates. Color development was stopped with 2 mol/L of H_2_SO_4_ and the absorbance was measured at 450 nm by Microplate Reader.

### SABC-IHC staining

The primary antibody is the polyclonal antibody made for this experiment and the secondary antibody is HRP conjugated goat anti-rabbit IgG.

Staining steps. The microsections were deparaffinated and washed; 3% H_2_O_2_ was added for approximately 15 min at room temperature, and the microsections were then washed with distilled water: 2 min×3 times; 0.1% trypsin was added for approximately 40 min at 37˚C, and the microsections were washed with distilled water: 2 min×3 times; 5% BSA was added for approximately 40 min at 37˚C without washing; The primary antibody (1:400 rabbit polyclonal antibody against Bactrian camel FcμR) was added to the positive group and allowed to stand for approximately 16 hours at 4˚C; for the negative group, PBS solution was added instead ofthe primary antibody and allowed to stand at 4˚C for approximately 16 hours; then, both were washed with PBS 5 min×4 times; secondary antibody (1:500 HRP conjugated goat anti-rabbit IgG) was added, and incubated at 37˚C for 40 min, followed by washing with PBS 5 min × 4 times; SABC was added and allowed to stand at 37˚C for 30 min, followed by washing with PBS 5 min × 4 times; DAB color rendering was performed at room temperature and away from light, the color rendering effect was observed under a light microscope, and the excess color rendering liquid was then washed away; The microsections were redyed with a small amount of hematoxylin dyeing solution, sealed with neutral balsam, and stored at 50˚C.

### Statistical analysis

Five slices were randomly selected for each lymph node of nine young healthy Bactrian camels, and ten fields of view were randomly selected for each slice for positive cell statistics (Image-Pro Plus). The number of cells was expressed as the average number of cells per high magnification field of view(400×). The obtained data were subjected to ANOVA using IBM SPSS software, and the results were expressed as "average value ± standard deviation", and make a table with OriginPro 2018C software. The difference in the number of positive cells among each group was analyzed by One-way analysis of variance, and the significant difference was indicated by P<0.05.

## Results

### Gene cloning and vector construction

**Fig 1:**
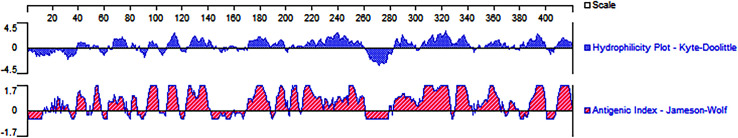
Prediction of hydrophilicity and epitope of FcμR protein in Bactrian camel.

**Fig 2:**
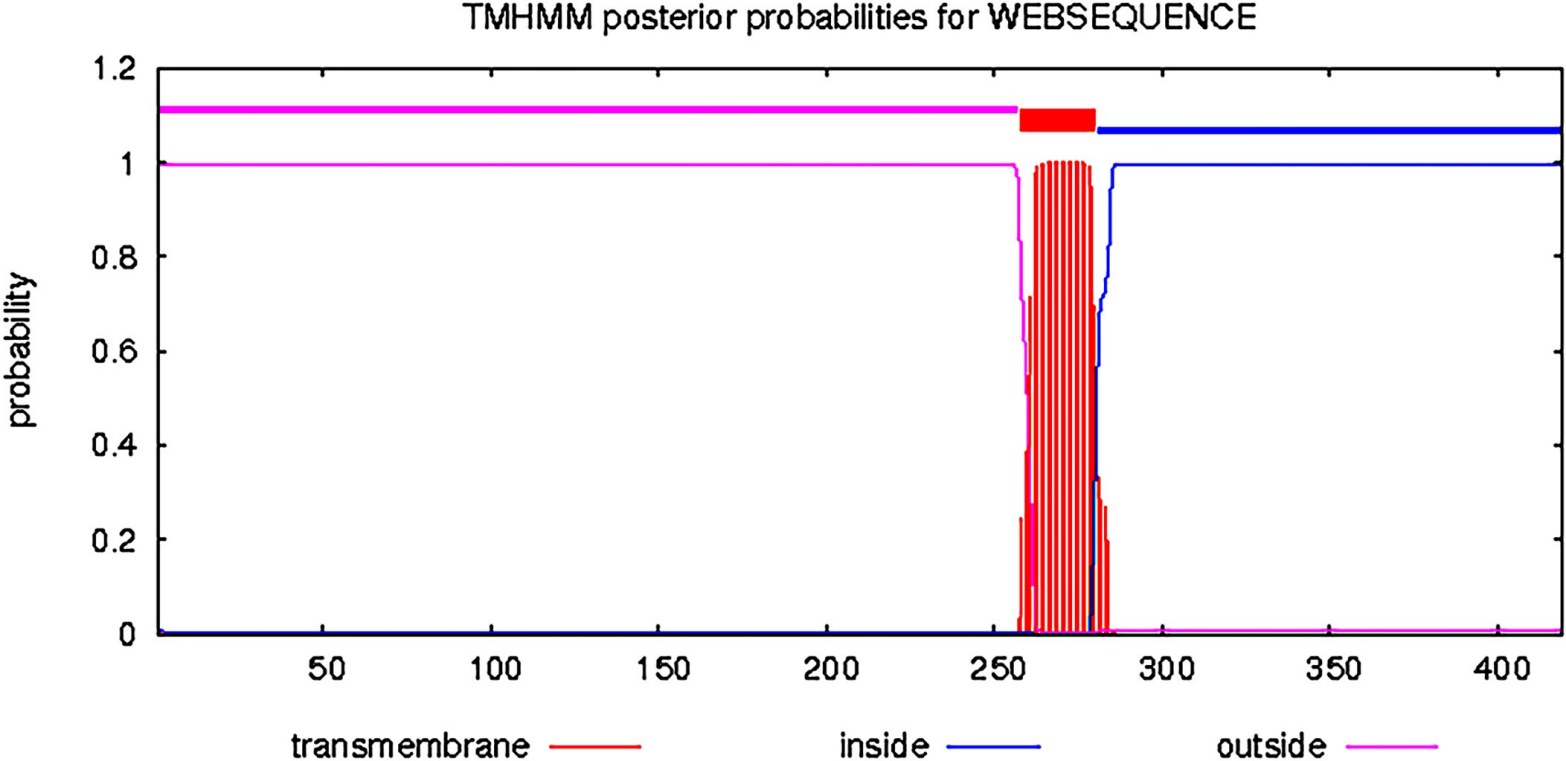
Prediction of FcμR protein transmembrane region in Bactrian camel.

**Fig 3:**
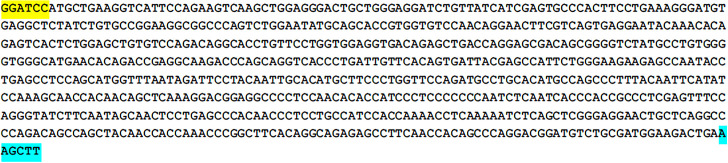
Synthesized gene sequences. 5’ add BamHI (Yellow) and 3’ add HindIII (Cyan).

**Fig 4:**
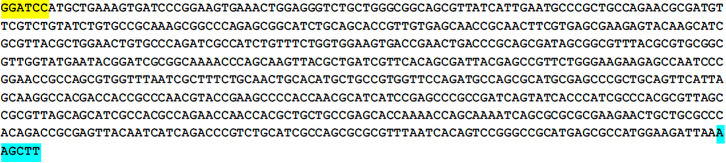
Codon optimized gene sequences. 5’ add BamHI (Yellow) and 3’ add HindIII (Cyan).

**Fig 5:**
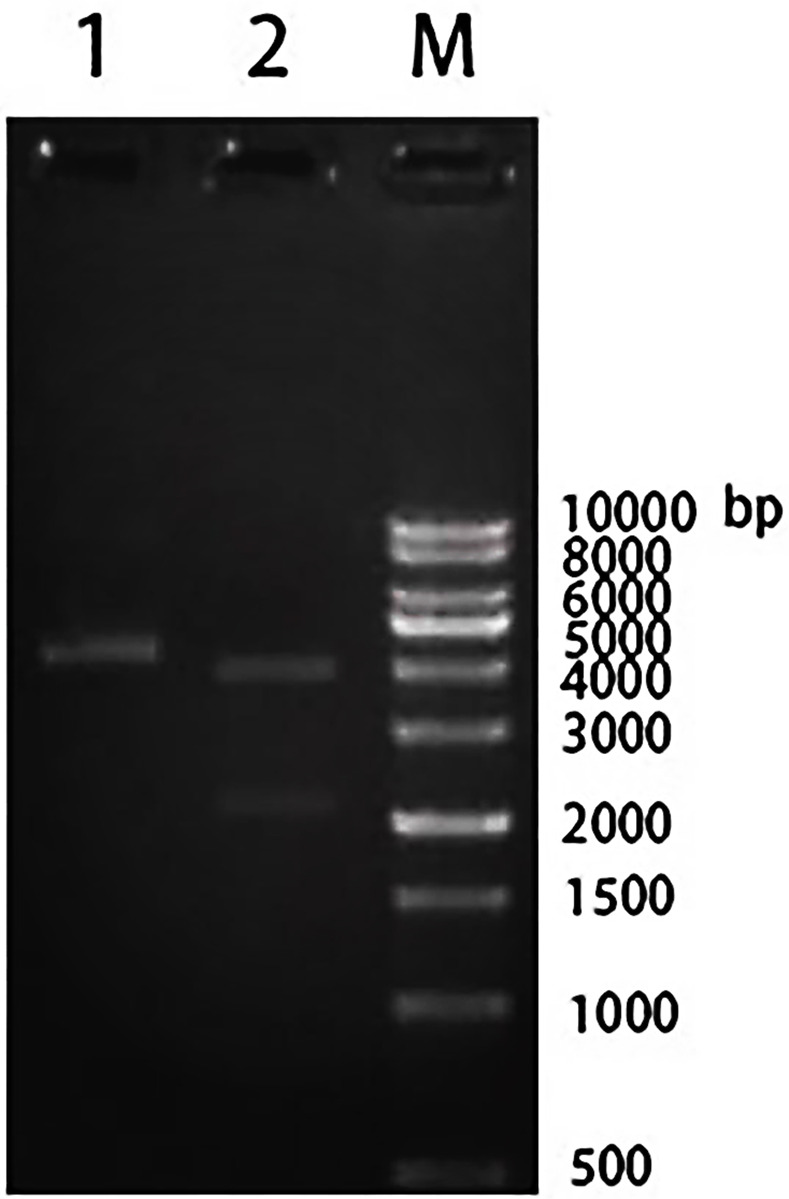
Enzymatic digestion of recombinant plasmids. 1.undigested 2.digested with EcoRV and Xhol M.ladder.

The Bactrian camel FcμR gene fragment protein selected in this paper has a high antigenic index and strong hydrophobicity, as shown in Fig 1. The Bactrian camel FcμR CDS region has a total of 419 amino acids, the 1-257th amino acid is the extramembrane region, the 258-280th amino acid is the transmembrane part, and the 281-419th amino acid is the intracellular region, as shown in Fig 2. The target gene sequence is shown in Fig 3. The optimized gene sequence is shown in Fig 4. The experimental results of restriction enzymatic digestion of recombinant plasmids reported by Suzhou GENEWIZ Biotechnology are shown in Fig 5.

### Induced expression of recombinant proteins

**Fig 6:**
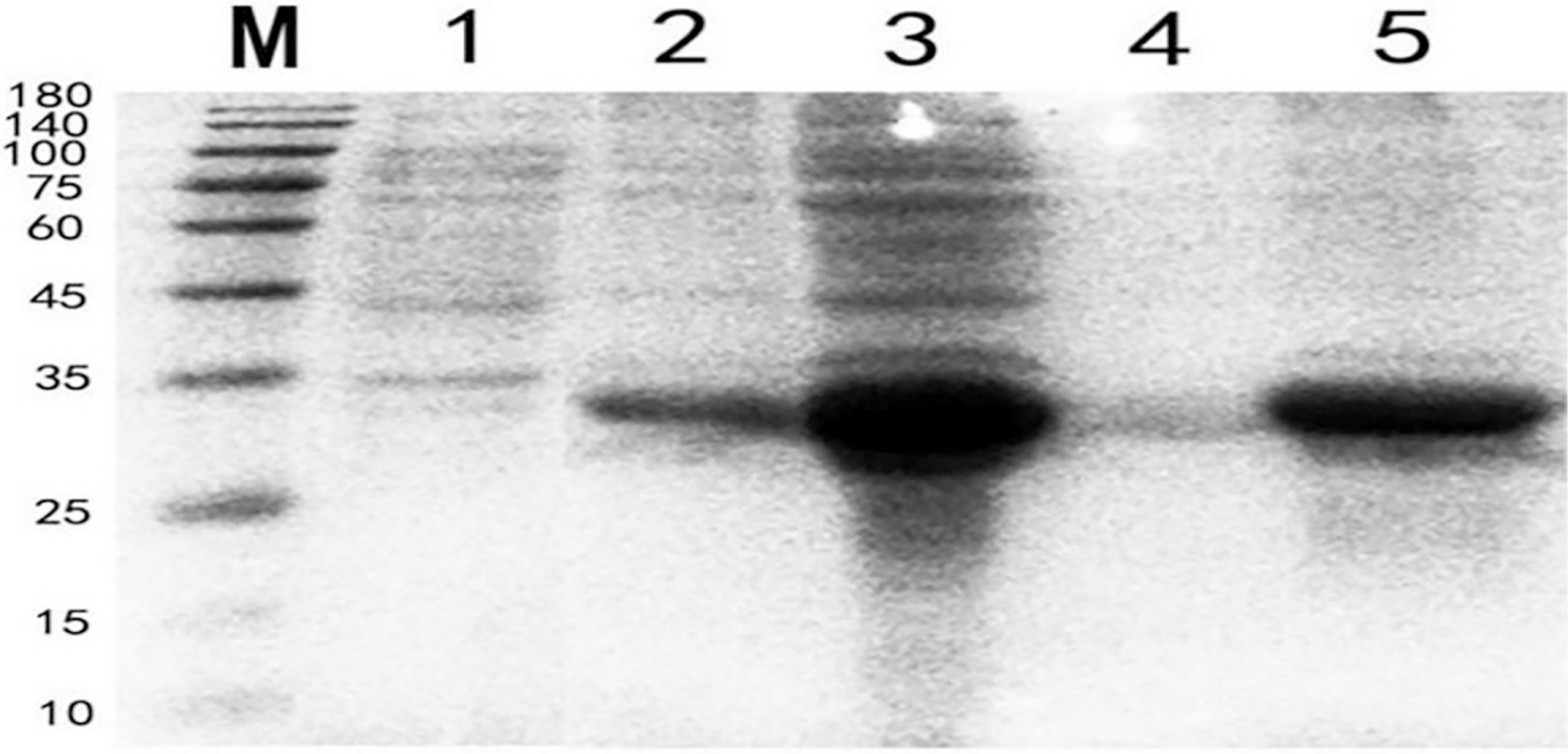
Expression and types of recombinant proteins. M.Protein molecular quality standard 1.Uninduced expression of recombinant proteins 2.Recombinant proteins that have been induced to express 3.The recombinant protein after breaking the wall 4.Supernatant 5.Sedimentation.

The supernatant, sedimentation were separated for electrophoresis on SDS-PAGE, which are shown in Fig 6.

### Optimization of recombinant protein expression conditions

**Fig 7:**
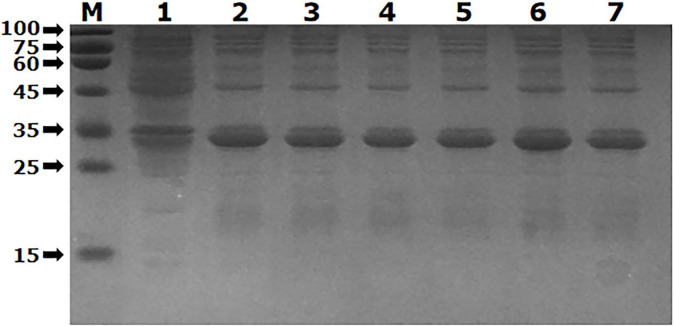
Optimal induced concentration of recombinant protein was determined. M. Protein molecular quality standard; IPTG of 0 μg/ml、0.1 μg/ml、0.3 μg/ml、0.5 μg/ml、0.7 μg/ml、0.9 μg/ml and 1.0 μg/ml were added in lanes 1–7, respectively.

**Fig 8:**
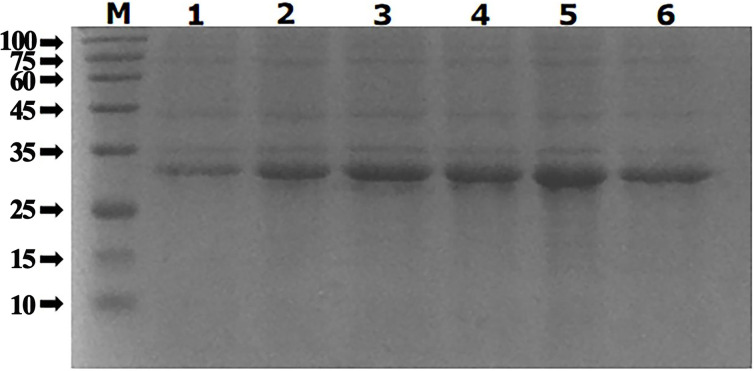
Optimal induction time for recombinant proteins. M. Protein molecular quality standard; The induction expression time of lane 1–6 was 1 h、2 h、3 h、4 h、5 h and 6 h respectively.

The recombinant protein was induced to express by setting different gradients of inducers and different induction times, and the results are shown in Figs 7 and 8.

### Potency detection of polyclonal antibodies

When the positive serum and the negative serum are diluted at 1:32000 and 1:2000 respectively, the OD tested/OD negative is ≥2.1, indicating that the serum titer of the rabbit anti-Bactrian camel FcμR recombinant protein is 1:32000, and the results are shown in Table 1.

**Table 1:** The titer detection of polyclonal antibody serum.

Dilution	OD_450_	P/N	+/-
1:2000	1.46	5.21	+
1:4000	1.22	4.36	+
1:8000	1.09	3.89	+
1:16000	0.80	2.86	+
1:32000	0.71	2.54	+
1:64000	0.38	1.36	-
1:128000	0.28	1	-
Control	0.26		
Control	0.28		

Effective Dilution: + Invalid Dilution: -

### Western blotting (WB)

**Fig 9:**
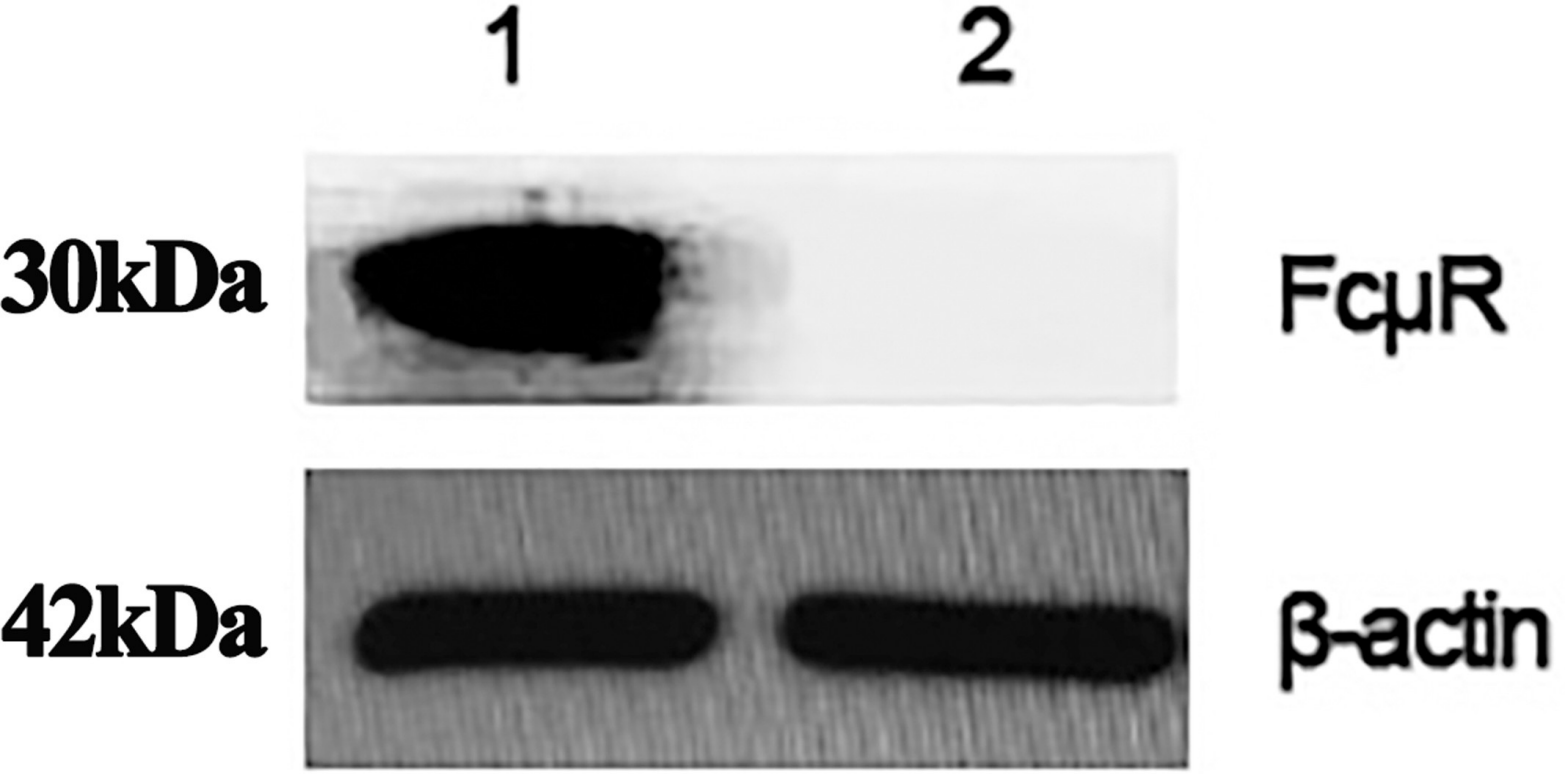
Results of western blotting. 1.Rabbit antiserum 2.Rabbit serum before immunization. Independent experiments were conducted three times with β-actin as the internal control.

There are obvious western blots at a size of about 30kDa, indicating that the polyclonal antibody we prepared has good immunogenicity, and the results are shown in Fig 9.

### Basic structure of lymph nodes in Bactrian camels

**Fig 10:**
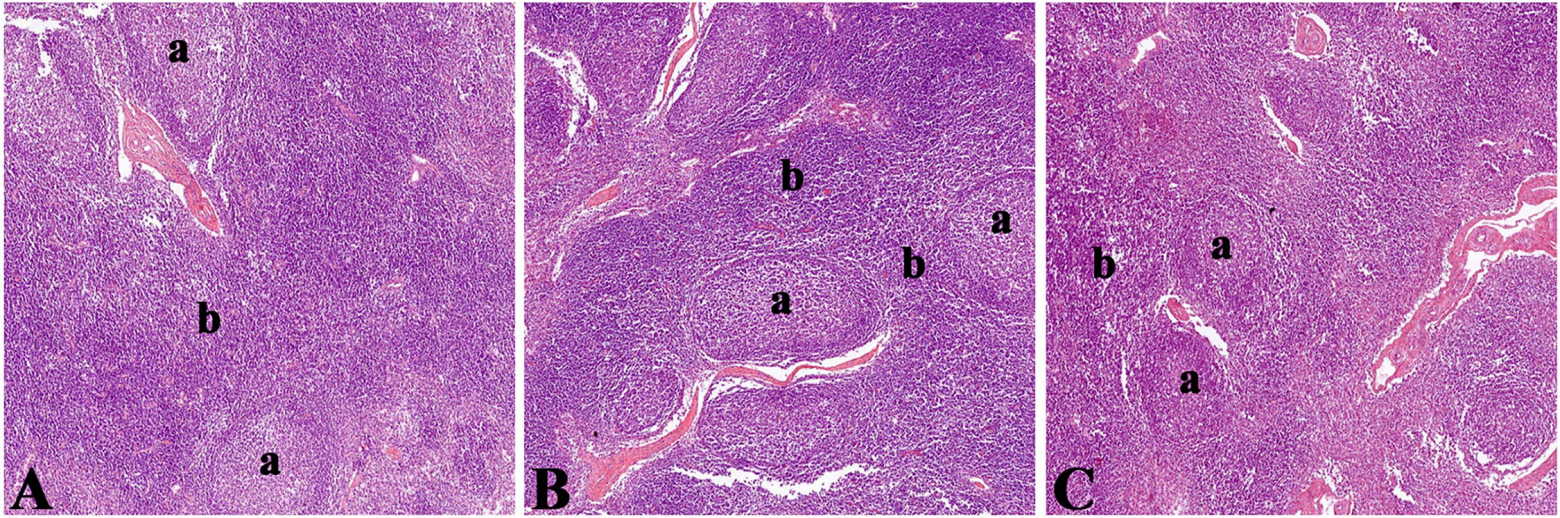
Tissue structure of small intestinal lymph nodes in Bactrian camel. A, B, and C represent duodenal, jejunal, and ileal lymph nodes, respectively; a lymphoid follicle; b interfollicular lymphoid tissue.

In order to understand the basic structure of the lymph nodes of Bactrian camels, H&E staining was performed on the small intestinal lymph nodes of Bactrian camels, and the staining results are shown in Fig 10.

### Distribution of FcμR positive cells in small intestinal lymph nodes of Bactrian camel

**Fig 11:**
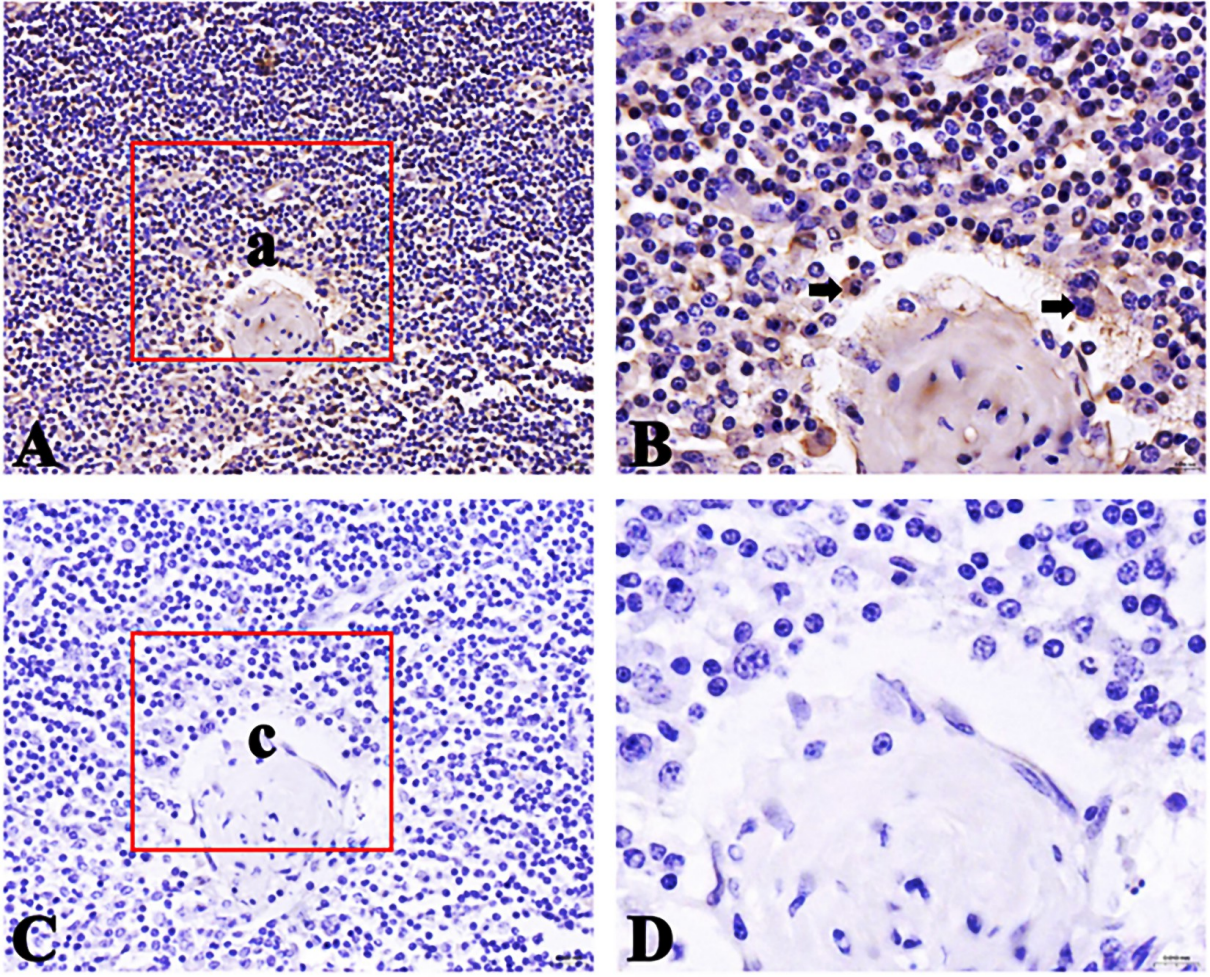
Distribution of FcμR positive cells in duodenal lymph nodes. A. Positive cells (40×); B Local amplification of a (100×); C negative control (40×); D Local amplification of c (100×); Positive cells are shown by arrows.

**Fig 12:**
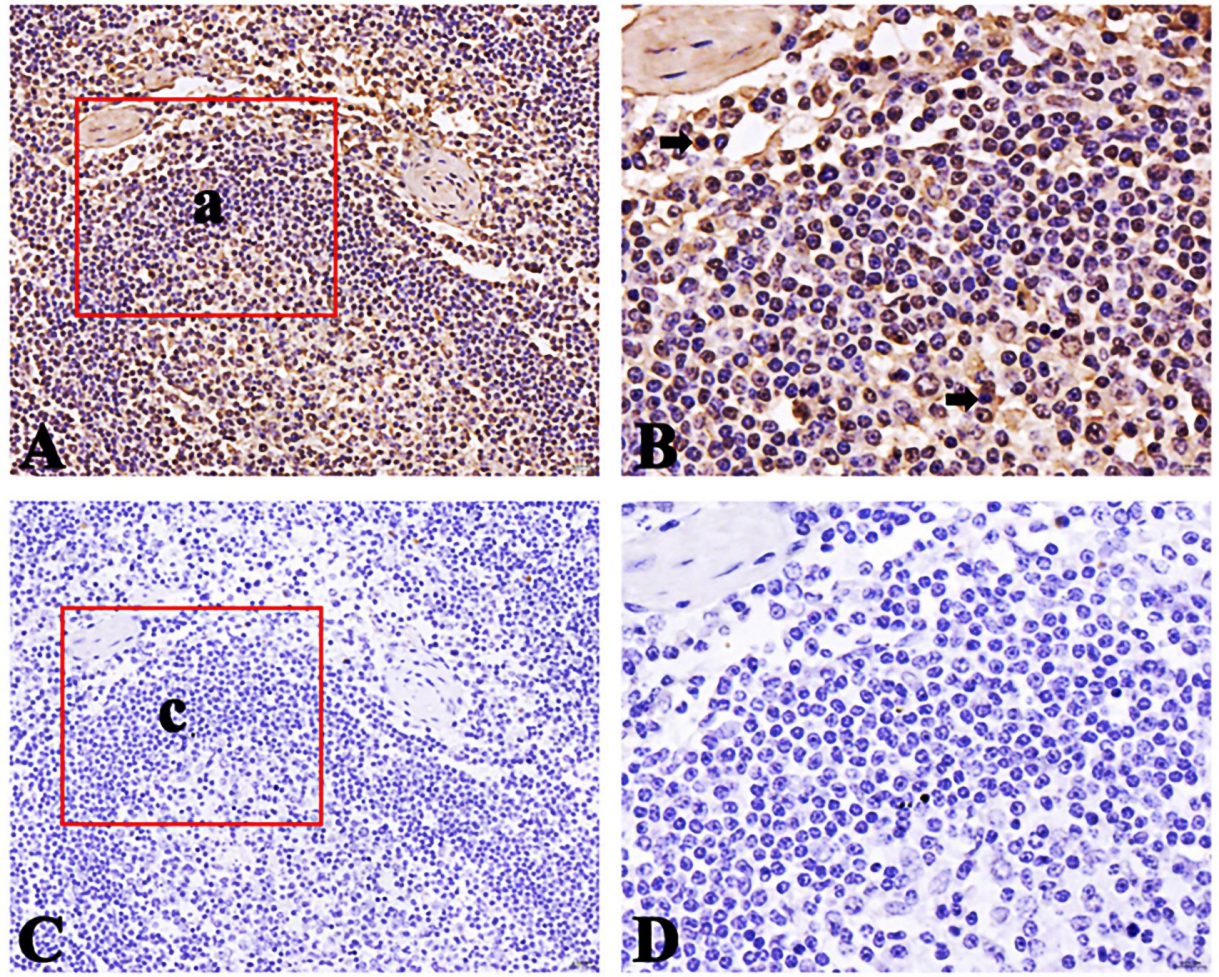
Distribution of FcμR positive cells in jejunum lymph nodes. A. Positive cells (40×); B Local amplification of a (100×); C negative control (40×); D Local amplification of c (100×); Positive cells are shown by arrows.

**Fig 13:**
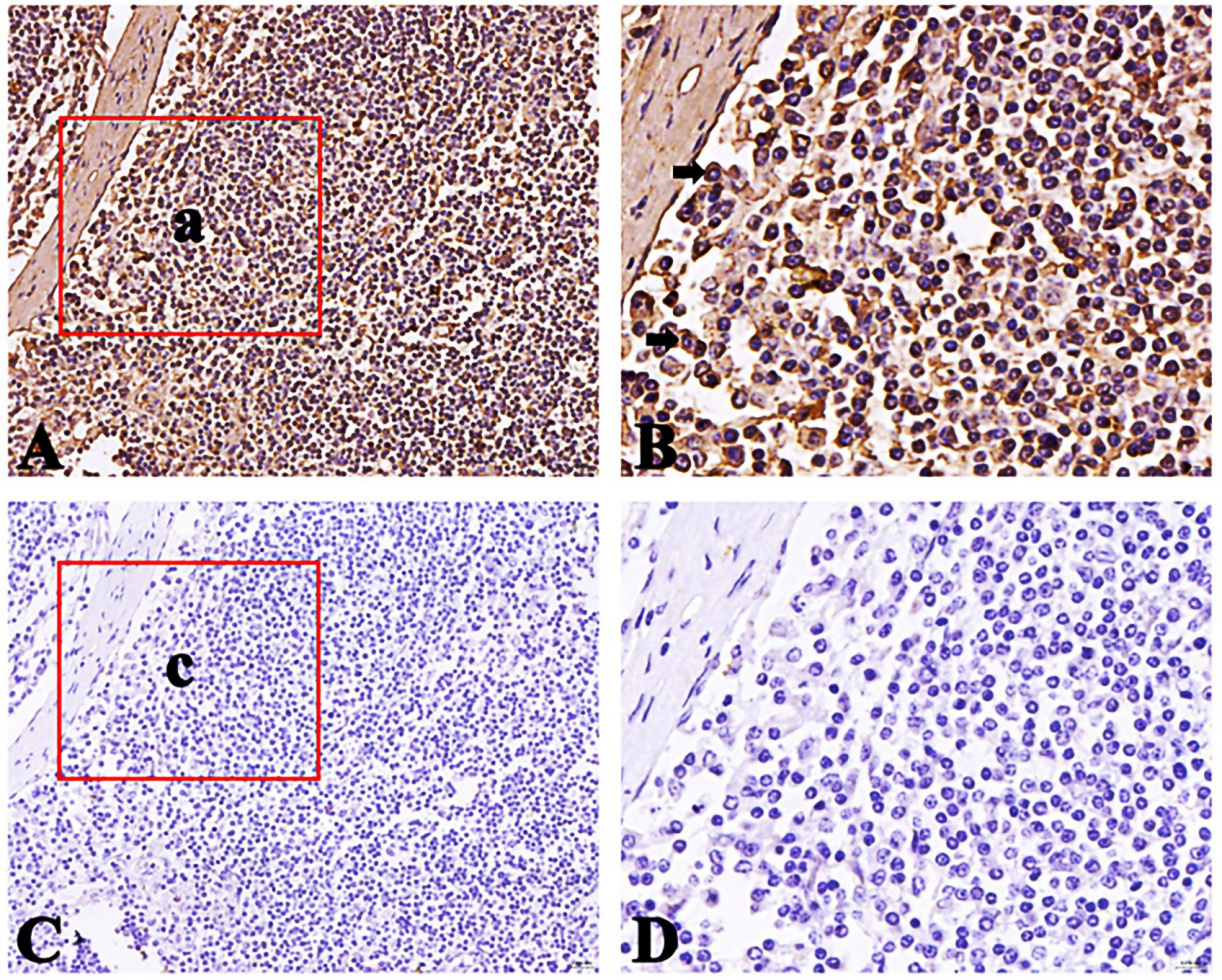
Distribution of FcμR positive cells in ileum lymph nodes. A. Positive cells (40×); B Local amplification of a (100×); C negative control (40×); D Local amplification of c (100×); Positive cells are shown by arrows.

The FcμR positive cells appear brownish-yellow and are mainly distributed in the lymphoid follicular area of lymph nodes. The FcμR positive cells are also distributed in the germinal center, but the number is less than that in the lymphoid follicle area. The results are shown in Figs 11–13.

### Expression of Bactrian camel FcμR positive cells in small intestinal lymph nodes

**Fig 14:**
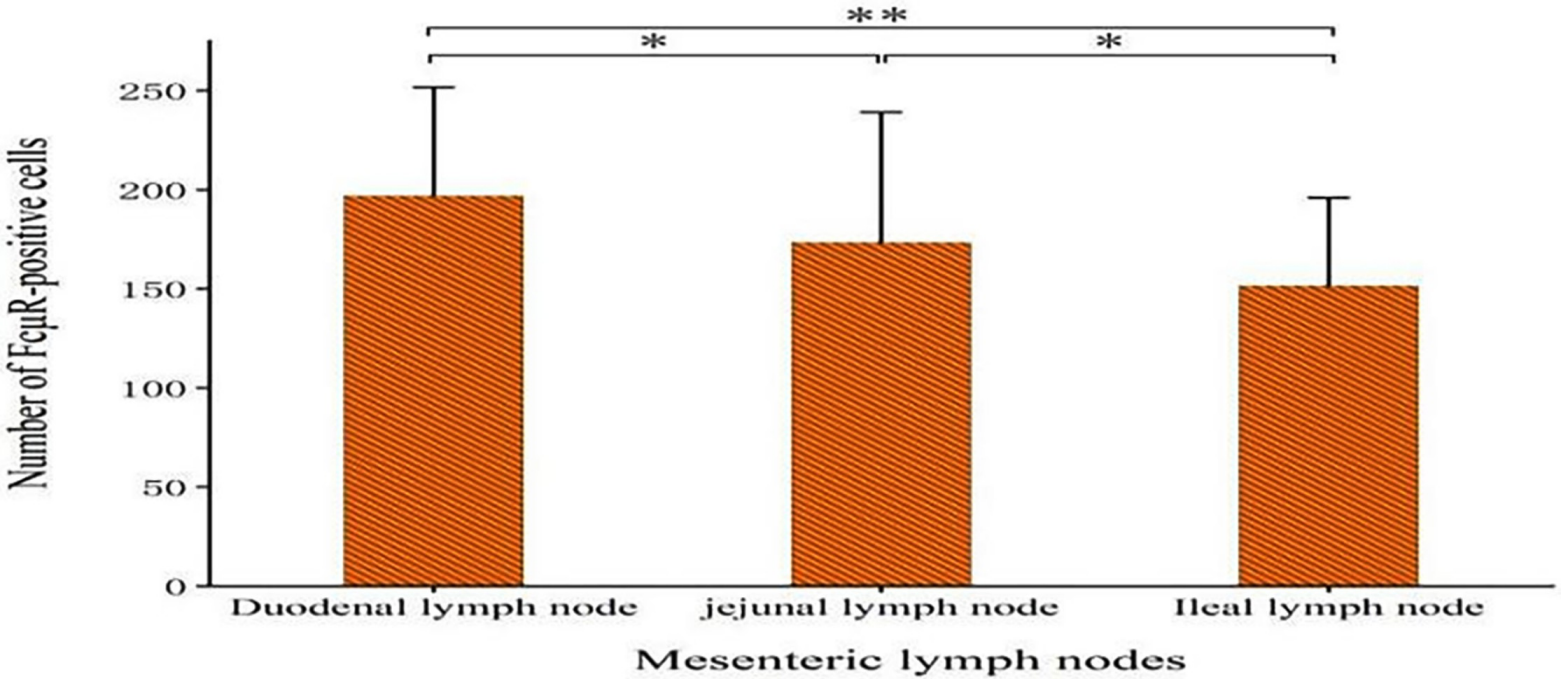
Expression of FcμR positive cells in small intestinal lymph nodes. Data represent the mean±SD.*P<0.05,**P<0.01.

**Fig 15:**
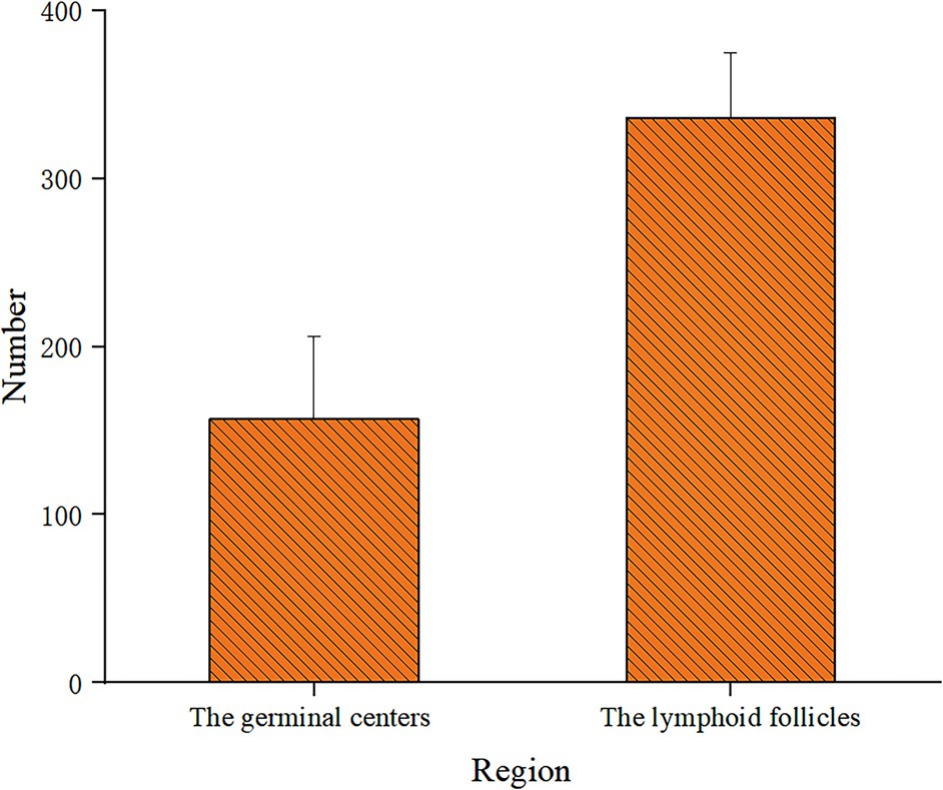
Expression of FcμR positive cells in different zones. Data represent the mean±SD.*P<0.05,**P<0.01.

We found that the number of FcμR positive cells decreased in the order of duodenal lymph nodes, jejunal lymph nodes and ileal lymph nodes, and the number of positive cells between different intestinal segments were statistically significant (P<0.05), the results are shown in Fig 14 (Significant difference is indicated by *, extremely significant difference is indicated by **). The number of FcμR positive B cells in the germinal centers of intestinal lymph node of Bactrian camels was much lower than that of lymphoid follicles, and the number of positive cells between different regions is statistically significant(P<0.05). The results are shown in Fig 15.

## Discussion

IgM is a member of the immunoglobulin family and is the first immunoglobulin to be secreted during the body’s initial immune response, accounting for 5–10% of total serum immunoglobulins [21, 22]. As an important participant in the body’s humoral immunity, IgM has the functions of clearing apoptotic cells, preventing infection and maintaining tissue homeostasis [23]. It is worth noting that the immunological function of IgM is mainly achieved through FcμR [14]. FcμR is the specific receptor of immunoglobulin IgM, and it is also a regulatory factor for the production of antibodies by TI antigen and TD antigen, which can promote the formation of germinal centers and reduce the threshold of BCR signal transduction [24]. This paper confirms for the first time that Bactrian camels are another newly discovered FcμR expression species after humans and mice, which greatly expands the research prospects of immunoglobulin IgM specific receptors. Although the small intestine of Bactrian camel is a whole, but there is a certain independence among the mesenteric lymph nodes. The number of FcμR positive cells in Bactrian camels decreased in the order of duodenal lymph nodes, jejunal lymph nodes and ileal lymph nodes, and the number of positive cells between different intestinal segments was statistically significant (P<0.05). The reason for this phenomenon might be due to duodenum being the main site of the body to absorb nutrients, and the material exchange at this site is very frequent. A large amount of nutrients and external pathogenic microorganisms will be transported to the lymph nodes, which stimulate the development and differentiation of local immune cells [25, 26]. Therefore, the immune cells in the duodenal lymph nodes are the most active, bear the greatest immune pressure, and store the largest number of immune cells. As the chyme moves backwards, the number and variety of pathogenic microorganisms begin to decrease, and this, combined with the reduced frequency of material exchange, leads to a decreasing trend in the number of FcμR positive cells in the small intestinal lymph nodes. Interestingly, Gourbeyre P [27] found that PRRs (Pattern recognition receptors) pattern recognition receptors and their related immune response mediators were more expressed in jejunum and ileum than in the duodenum, indicating that the jejunum lymph nodes and ileal lymph nodes are the main production sites of immune cells in the small intestine. The main production sites of small intestinal immune cells were observed in jejunum and ileum, while the main effect site of small intestinal immune cells was in the duodenum. The separation of the immune cell production site and the effect site can not only ensure the unity of duodenal absorption and immune defense, but also significantly improve the work efficiency of the entire small intestine, which is of great significance to maintaining the health of the body.

As an important part of the peripheral immune system, lymph nodes play a pivotal role in the process of local mucosal immune response. Bactrian camel lymph nodes are mainly composed of lymphoid follicles and interfollicular lymphoid tissue, which were mixed with each other and scattered throughout the lymph nodes. This similar structure was observed in lymph nodes of dromedary as reported by Mohamed Zidan [28], which was similar to those of pigs [29], but different from that of humans [30], cattle [31] and buffalo [32]. As we all know, the anatomical structure of a typical lymph node is mainly composed of cortex and medulla, and the cortex can be further divided into lymphoid nodules and paracortical areas, which are the main areas for the activation and differentiation of immune cells induced by external antigens. The anatomical structure of the Bactrian camel’s lymph node is equivalent to many typical lymph node cortices are spliced together. So it has a stronger immunological function, which may be one of the important reasons why Bactrian camels can tolerate extreme conditions and maintain their health. We also found that the number of FcμR positive B cells in the germinal centers of intestinal lymph node of Bactrian camels was much lower than that of lymphoid follicles marginal zone, and the number of positive cells between different zones is statistically significant (P<0.05). There are three main reasons for this discrepancy: First, the lymphoid follicle marginal zone is located at the interface of blood, lymph fluid, and lymphoid tissue, and is the entrance for external antigens to enter the lymph node. FcμR positive cells located in the marginal zone of lymphoid follicles can not only contact external antigens for the first time to induce host cell activation, but also act as a local immune defense barrier [16]. Second, FcμR molecules are mainly expressed in B cells, and the FcμR molecules on the surface of germinal center B cells is significantly lower than that of lymphoid follicle marginal zone B cells, so the number of FcμR positive cells in germinal center will be less than that in lymphoid follicle marginal zone FcμR positive cells, indicating that FcμR molecules may have negative effects on activated B cells [9]. Third, the surface of mature plasma cells does not express FcμR molecules. Therefore, As B cells differentiate into plasma cells, the number of FcμR positive cells in the germinal center is objectively reduced. In summary, the number and distribution sites of FcμR positive cells are not only closely related to the immunological function of small intestinal mesenteric lymph nodes, but also has constructive significance for the construction of peripheral mucosal immune system.

## Conclusion

This paper confirmed for the first time that Bactrian camel is a newly discovered expression species of FcμR, which greatly expanded the research scope and research prospects of FcμR. Bactrian camel lymph nodes were mainly composed of lymphoid follicles and interfollicular lymphoid tissue, the same as dromedary lymph nodes, but different from typical lymph nodes. The number of FcμR positive cells decreased in the order of duodenal lymph nodes, jejunal lymph nodes and ileal lymph nodes, and the number of positive cells was statistically significant between different intestinal segments (P<0.05). The number of FcμR positive B cells in the germinal centers of intestinal lymph node of Bactrian camels was much lower than that of lymphoid follicles marginal zone, and the number of positive cells between different zones is statistically significant (P<0.05).

## Supporting information

S1 Raw images (PDF)
