# Supplementary figures and images for: Distribution characteristics of FcμR positive cells in small intestinal lymph nodes of Bactrian camel
Source: PLoS One. 2023 Jul 20;18(7):e0287329. doi: 10.1371/journal.pone.0287329 (PMC10358951; doi:10.1371/journal.pone.0287329)

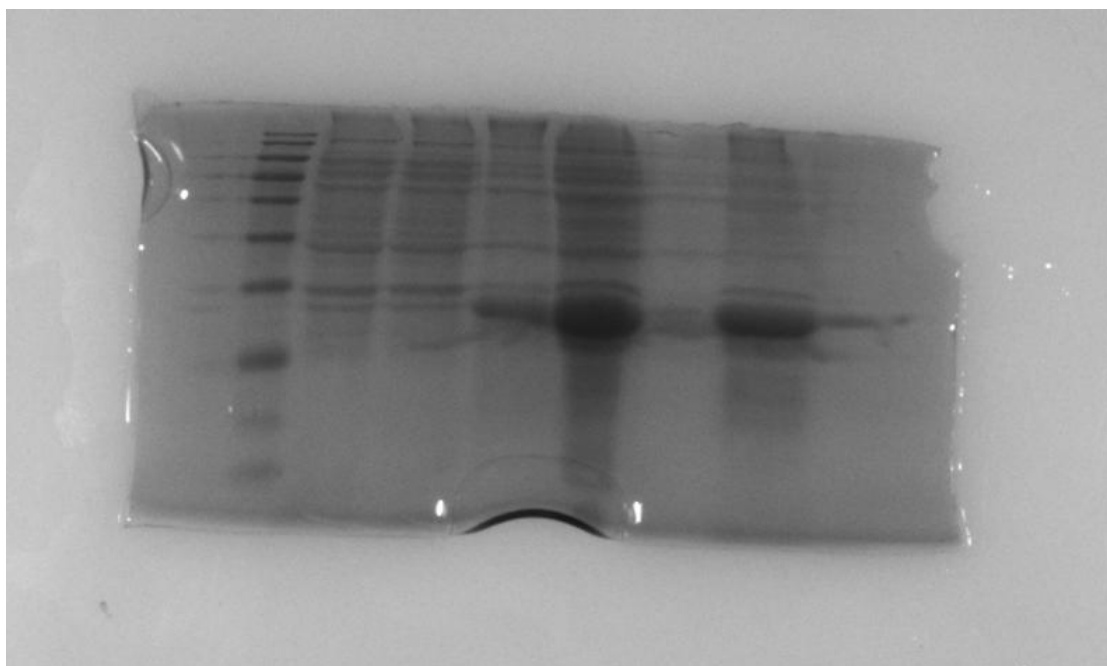

S6\_Fig

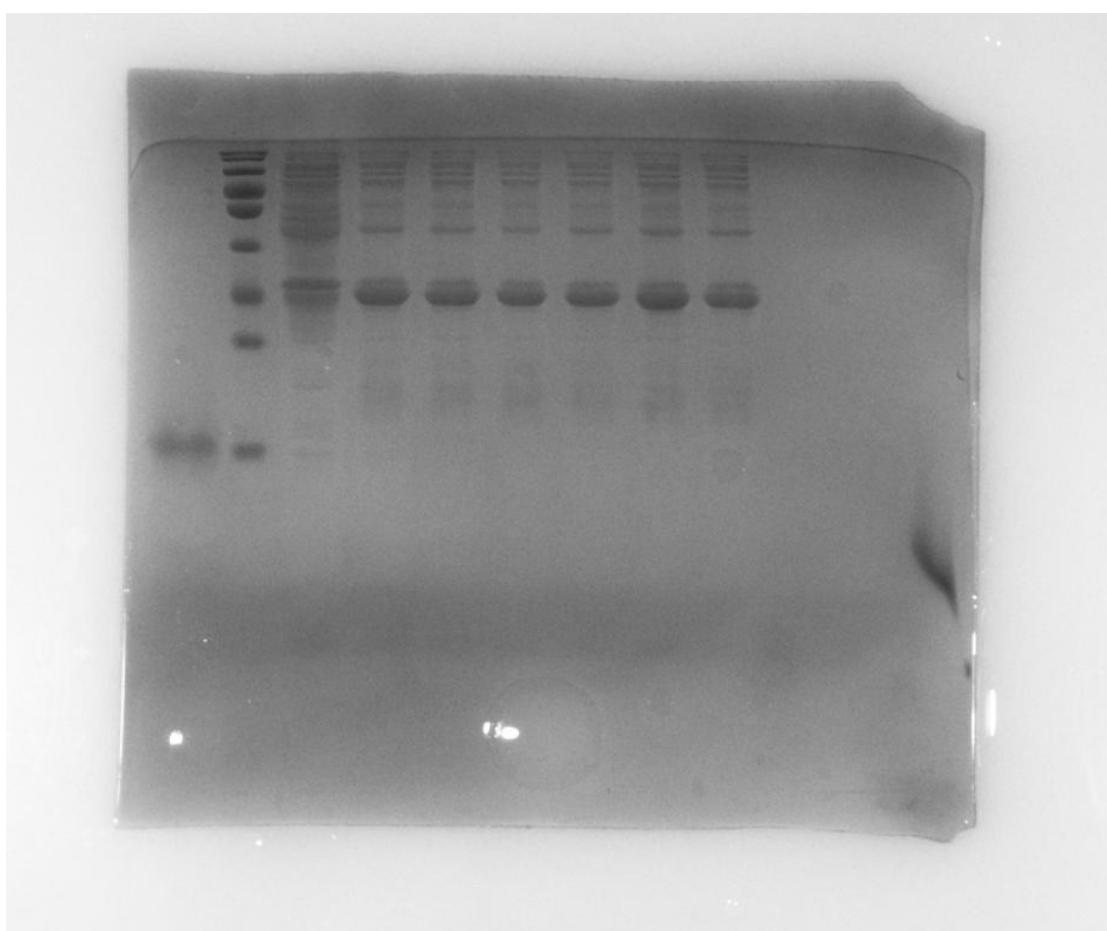

S7\_Fig

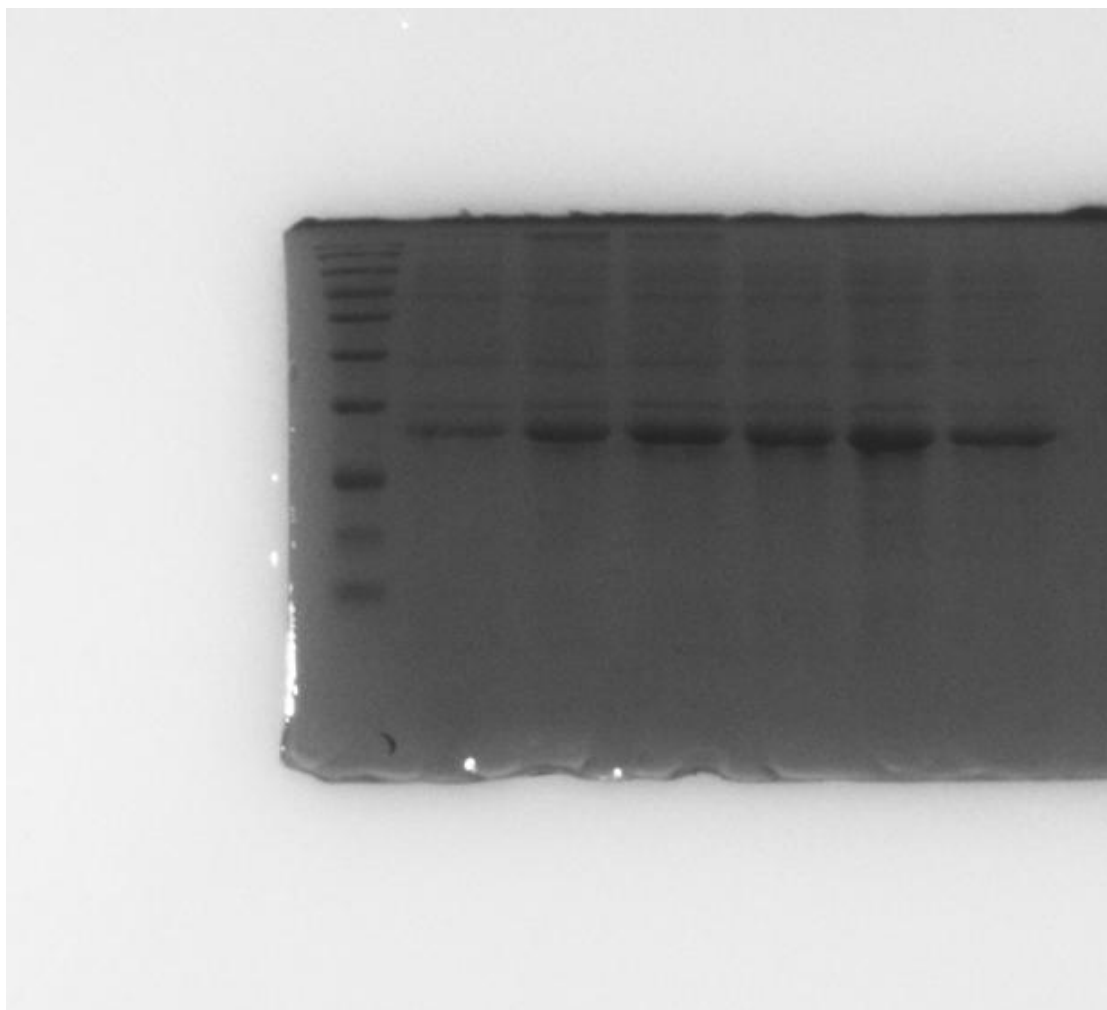

S8\_Fig

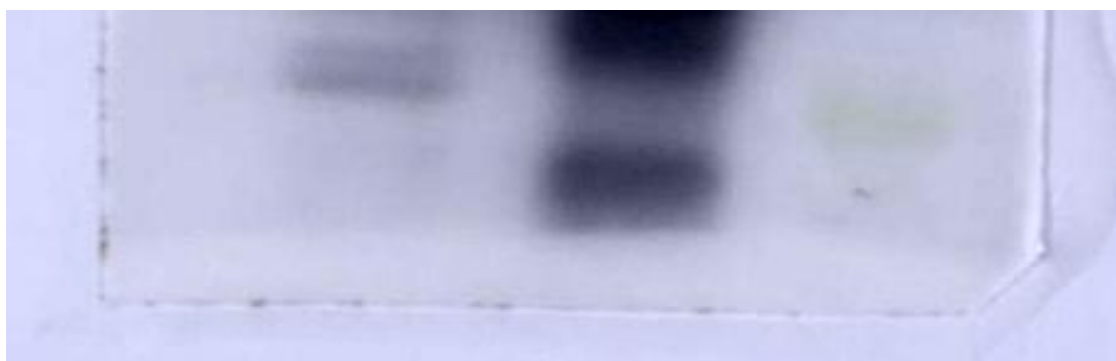

S9\_Fig

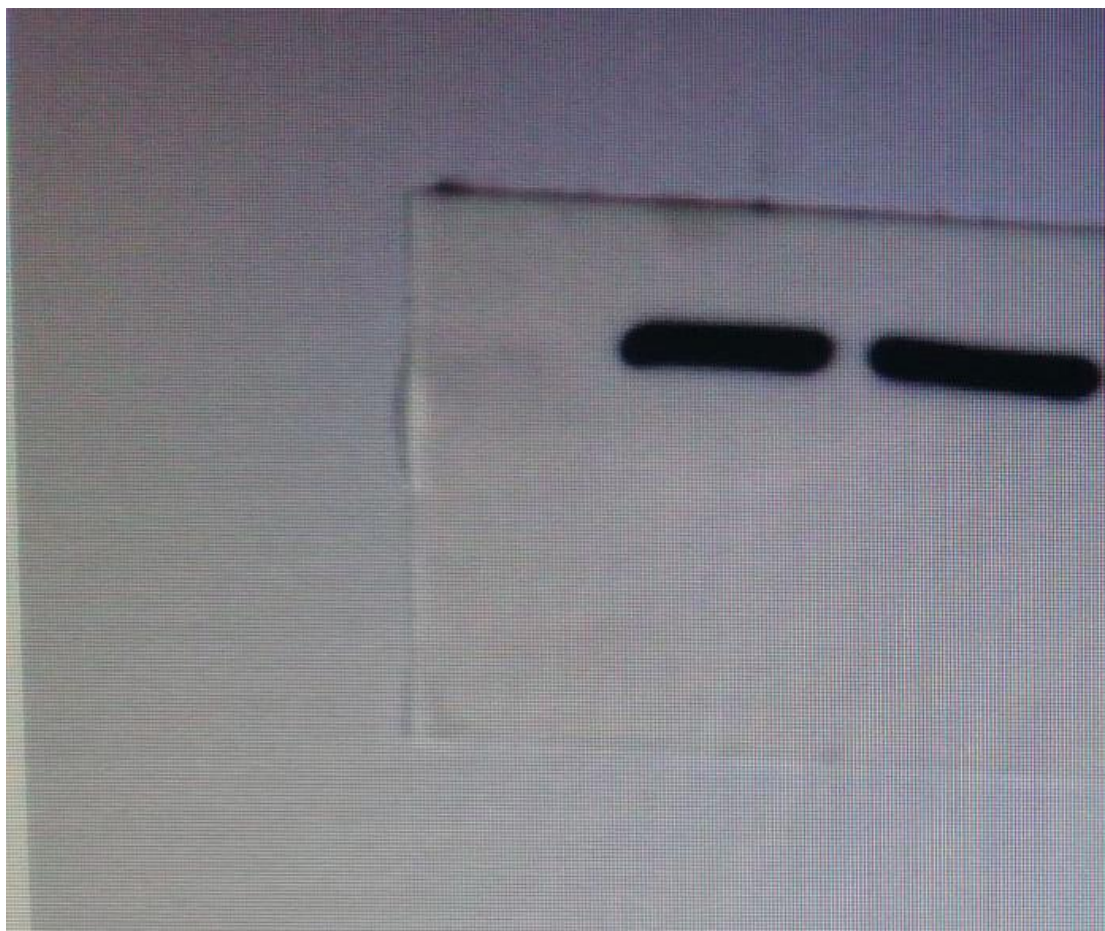

$\beta$ -actin

Supplement: S1 Raw images — (PDF) [file pone.0287329.s001.pdf]
